# The Effect of Yttria Content on Microstructure, Strength, and Fracture Behavior of Yttria-Stabilized Zirconia

**DOI:** 10.3390/ma15155212

**Published:** 2022-07-28

**Authors:** Volodymyr Kulyk, Zoia Duriagina, Andrii Kostryzhev, Bogdan Vasyliv, Valentyna Vavrukh, Olexandra Marenych

**Affiliations:** 1 Department of Materials Science and Engineering, Lviv Polytechnic National University, 12 S. Bandera str., 79013 Lviv, Ukraine; zduriagina@ukr.net (Z.D.); vavrukh.valentyna@gmail.com (V.V.); 2 Department of Materials Engineering, John Paul II Catholic University of Lublin, 14 Racławickie Al., 20-950 Lublin, Poland; 3 Centre for Microscopy and Microanalysis, University of Queensland, St. Lucia, Brisbane, QLD 4072, Australia; a.kostryzhev@uq.edu.au (A.K.); o.marenych@uq.edu.au (O.M.); 4 Department of Hydrogen Technologies and Alternative Energy Materials, Karpenko Physico-Mechanical Institute, 5 Naukova str., 79060 Lviv, Ukraine; mechengin1111@gmail.com

**Keywords:** YSZ ceramics, microstructure, porosity, grain morphology, element distribution, phase composition, flexural strength, fracture micromechanism

## Abstract

Yttria-stabilized zirconia (YSZ) is well-known as a material with perfect mechanical, thermal, and electrical properties. It is used for manufacturing various high-temperature components for aerospace and energy generation, as well as wear- and corrosion-resistant devices in medicine. This work investigated the effect of a Y_2_O_3_ addition to ZrO_2_ on the microstructure and mechanical properties of YSZ ceramics produced by one sintering schedule. ZrO_2_ ceramics doped with 3, 4, 5, 6, 7, and 8 mol% Y_2_O_3_ (designated 3YSZ through to 8YSZ) were prepared by using conventional sintering at 1550 °C for 2 h in argon. The effect of yttria content was analyzed with respect to grain size, morphology of the microstructural features, phase composition, parameters of fracture surface, and flexural strength. The 7YSZ ceramics sintered at 1550 °C for 2 h showed the highest level of flexural strength due to the formation of the fine-grained microstructure containing mainly the monoclinic and tetragonal zirconia phases. The fracture micromechanism in the studied YSZ ceramics is discussed.

## 1. Introduction

ZrO_2_-based ceramics are widely used in biomedical applications (particularly for teeth crowns, bridges, implant fixtures, and dental prostheses) [1,2,3], energy generation [4,5], chemical industry [6,7], and the manufacturing of various machine components [8,9,10,11]. Doping with Y_2_O_3_ improves strength, toughness, and wear resistance [12], thus opening a possibility of application in orthopedics for hip-joint heads [13,14].

The required strength and toughness are achieved via grain refinement of the tetragonal crystal structure [15]. Under stress, the tetragonal phase can strengthen via the tetragonal–monoclinic transition near the crack tip. Thus, the energy dissipation occurs at the crack tip, leading to the crack growth retardation. The crystal structure of zirconia is monoclinic at room temperature and atmospheric pressure. Zirconia undergoes a phase transformation during heating from monoclinic to tetragonal (at 1173 °C) and to cubic (at 2370 °C). High-temperature phases are unstable at 20 °C, although they can be stabilized by alloying with CaO [16], MgO [17], Y_2_O_3_ [14,18], and CeO_2_ [19]. In stomatology, for example, the tetragonal phase is preferred due to the combination of high strength and toughness, and optimum optical properties. The phase stabilization is usually achieved with Y_2_O_3_ doping.

Transformation hardening in ZrO_2_ ceramics stabilized with 3–4 mol% Y_2_O_3_ depends on the grain size [20]. In a microstructure with submicron-sized grains, spontaneous tetragonal–monoclinic transformation does not take place [20]; consequently, softening does not follow. In Reference [21], the authors suggested that an increase in strength of 5 wt.% Y_2_O_3_-stabilized ZrO_2_ to 1540 MPa could be related to a significant (9.5%) fraction of the monoclinic phase observed after sandblasting. In stomatology, this kind of treatment improves the adhesion of cement to the core material. Sandblasting creates compression stresses on the surface, increasing the average bending strength of the zirconia ceramics [21]. Thus, grains of the tetragonal phase may transform to monoclinic not only due to alloying with various oxides, but also due to external stresses associated with grinding and sandblasting.

In References [22,23,24,25], the authors studied zirconia doped with 5.2 wt.% Y_2_O_3_ and 0.25 wt.% Al_2_O_3_. The microstructure in this material consisted mainly of tetragonal grains, although areas of the cubic phase were observed inside some tetragonal grains. These areas of the cubic phase not only did not retard the growth of the surrounding tetragonal phase but also grew along with it during heating at the sintering temperature of 1500 °C for 2 h [22,23,24,25].

The relative density of ZrO_2_ with 3 mol% Y_2_O_3_ increased with the temperature and holding time and reached 95% and 99% after sintering for 50 h at 1300 and 1500 °C, respectively [26]. The average grain size also increased with an increase in temperature and holding time and was in the range of 0.2–0.9 μm after holding for 50 h in the temperature range of 1300–1500 °C. During holding for 10 h, the fraction of the cubic phase grew from 11 to 15 wt.% at 1300 °C and from 15 to 19 wt.% at 1500 °C. After that, no significant variation in the phase fraction was observed [26].

With an increase in grain size, the zirconia becomes less stable and may exhibit spontaneous tetragonal–monoclinic transformation, not only at the crack tips, but through the whole volume [15]. This may lead to softening. The size and morphology of microstructural features (grains, pores, and grain boundaries) significantly depend on the sintering mode. A three-point bend testing of ZrO_2_ sintered at different temperatures and time periods showed a maximum strength of 904 MPa for a sintering temperature of 1580 °C [15]. However, SEM imaging of the microstructure did not reveal a significant variation in the grain size of ZrO_2_ sintered at 1510 °C for 120 min and at 1580 °C for 10 min.

With an increase in the sintering temperature and holding time, the grains grow and the number of micropores increases. Thus, after microwave sintering at 1500 °C for 20 min, the average grain size was 347 nm, and after traditional sintering at the same temperature of 1500 °C, but with a longer holding time of 40 h, the grain size was 1512 nm. This decreased the strength of zirconia stabilized with 3 mol% Y_2_O_3_ [2].

In Reference [19], zirconia was stabilized by CeO_2_. Following the plasma arc sintering at 2000 °C, a coarse-grain microstructure was formed with an average grain size of ~100 μm. High-temperature sintering in a vacuum reduced the oxygen content and facilitated the tetragonal–cubic transformation and grain growth.

The final sintering temperature, technological process, and heating method influence strength, porosity, and grain size of zirconia [27]. After sintering at 1300 °C, the bending strength of zirconia was 970 MPa, and after this, at 1500 °C, it was 1281 MPa, which was the maximum; moreover, after sintering at 1700 °C, the strength was 586 MPa. This can be related to grain growth in the temperature range of 1300–1700 °C. Above 1600 °C, not only the grain growth rate increases but the density of pores also increases.

ZrO_2_ with 5% Y_2_O_3_, <2% HfO_2_, and <1% (Al_2_O_3_ + SiO_2_) exhibited the smallest grain size of 0.07 μm, while the largest grain size of 0.35 μm was in ZrO_2_ alloyed with 4–6% Y_2_O_3_, <1% Al_2_O_3_, max 0.02% SiO_2_, max 0.01% Fe_2_O_3_, and max 0.04% Na_2_O [28]. This difference correlated with the sintering temperature: 0.07 μm was after sintering at 1350 °C, and 0.35 μm after this at 1600 °C.

In a number of works [2,29], the fine-grained ceramics showed better mechanical properties and a slower tendency for low-temperature degradation of the microstructure in water vapor. This is related to the retardation of the tetragonal–monoclinic transformation.

The fracture toughness in fully stabilized ZrO_2_ with 8 mol% Y_2_O_3_ was 3 MPa$\sqrt{m}$, and this, in partially stabilized ZrO_2_ with 3 mol% Y_2_O_3_, was 8 MPa$\sqrt{m}$ [29]. Although the variation in strength with Y_2_O_3_ was not studied in Reference [29], a lower toughness in more highly alloyed ceramics might be related to its higher strength and lower plastic properties.

The flexural strength of ZrO_2_(Y_2_O_3_)-20 wt.% Al_2_O_3_ was measured to be in the range of 760–1100 MPa, with a parabolic dependence on grain size [30]. The maximum strength of 1100 MPa was reached for a grain size of 1 μm. However, the fracture toughness decreased almost linearly from about 11 to 8 MPa$\sqrt{m}$ with an increase in grain size from 0.5 to 3 μm. Obviously, the optimum properties balance of ZrO_2_(Y_2_O_3_)-20 wt.% Al_2_O_3_ can be achieved via grain size selection. For this ceramic sintered at 1550 °C, the relative density was 99.3%, with an average grain size <0.9 μm. However, a further density increase to 99.6%, associated with an increase in the sintering temperature to 1650 °C, coincides with the grain-size growth up to 3 μm. The average grain size in this ceramic increased with an increase in the sintering temperature from 0.27 μm at 1450 °C to 1.28 μm at 1650 °C [30].

In Reference [31], the authors studied the effect of the concentration of zirconium dioxide on the strength and crack-growth resistance of Al_2_O_3_–ZrO_2_ ceramics. The phase composition of the studied specimens included α-Al_2_O_3_, as well as the monoclinic and tetragonal ZrO_2_ phases. The fraction of the tetragonal phase decreased with an increase in the total ZrO_2_ amount. The authors showed that additions of 10–20% ZrO_2_ efficiently inhibited the growth of Al_2_O_3_ crystals and, thus, positively affected the mechanical behavior of the ceramics. In Reference [32], the authors have proved that the optimal mechanical properties of zirconia ceramics and alumina doped with zirconia should be determined by estimating both the fracture toughness and strength characteristics.

The propensity to brittle fracture of YSZ ceramics stabilized by the various amounts of yttria was evaluated in References [33,34], based on the study of phase composition, microstructure, and fracture micromechanisms. Among YSZ ceramics containing from 3 to 5 mol% Y_2_O_3_, 5YSZ ceramics sintered at 1450 °C for 2 h exhibited the highest fracture toughness. The fracture toughness of this material was found to correlate with a high percentage of the tetragonal ZrO_2_ [33]. By comparing the mechanical behaviors of YSZ ceramics containing from 6 to 8 mol% Y_2_O_3_, it was found that 7YSZ ceramics sintered at 1600 °C for 2 h have the highest level of fracture toughness [34]. The above results are consistent with the mechanical characteristics of yttria-stabilized zirconia presented in Reference [35].

With an increase in sintering temperature, the ZrO_2_ grain size was found to grow, and the flexural strength decreased [27]. Therefore, the sintering temperature should be restricted to below 1550 °C. In this work, we investigated the effects of yttria content on the microstructure (grain size, pores density, and phase balance), flexural strength, and fracture characteristics (size and number density of fracture voids and cleavage facets) of YSZ ceramics sintered at 1550 °C. Micromechanisms of fracture development are discussed for various types of microstructures that are characteristic for YSZ ceramics.

## 2. Materials and Methods

The materials studied in this work were yttria-stabilized zirconia (YSZ) ceramics sintered from commercial starting powders. The zirconia and yttria powders were produced at the Vol’nogorskii Mining and Smelting Plant, Vol’nogorsk, Ukraine. Particle sizes were in the range of 100–150 nm for ZrO_2_ and 10–30 nm for Y_2_O_3_ powder. Series of square bar specimens of YSZ ceramics stabilized with 3, 4, 5, 6, 7, and 8 mol% Y_2_O_3_ (hereinafter 3YSZ to 8YSZ), approximately 4.2 mm × 4.2 mm × 50 mm in size, were sintered at 1550 °C, for 2 h, in a furnace with argon atmosphere. A detailed description of powder mixture preparation and sintering was given elsewhere [33]. To avoid phase transformations, the sintered specimens were slowly polished by using a metallographic polishing machine. In such a way, the required surface quality was reached.

The flexural tests of the bar specimens under three-point bend loading were carried out on an MTS Criterion E43.104 testing machine (MTS Systems Corporation, Eden Prairie, MN, USA) at 20 °C in air. The distance between the supporting rollers of the loading unit was 14 mm. Five specimens were tested for each material variant, and these were used to calculate the average values of fractural strength. For calculating the flexural strength of the material, the following formula was used [36]:

(1) $$\sigma_{f}=1.5\frac{Ps}{bh^{2}}$$

where *σ_f_* is flexural strength (MPa), *P* is the fracture load (N), *s* is the span between the supporting rollers (mm), and *b* and *h* are the width and height (mm) of a rectangular bar specimen, respectively.

Sample preparation for imaging and chemical analysis was carried out by using standard preparation techniques, including cutting with a diamond-coated metal disk on Struers Accutom 50, cold mounting in epoxy resin, and polishing on a Struers Tegramin machine (Struers, Copenhagen, Denmark). No sample etching or coating was used.

The microstructure and fracture surface characterizations were carried out by using an Olympus DSX1000 optical microscope (Olympus, Tokyo, Japan), Hitachi SU3500 scanning electron microscope (SEM) (Hitachi, Tokyo, Japan) equipped with Oxford energy dispersive X-ray spectroscopy (EDS) system and Aztec software (Oxford Instruments, Abingdon, UK), and Hitachi SU3900 SEM with Bruker EDS and Esprit software (Bruker, Billerica, MA, USA). Up to 370 pores, 530 grains, 300 fracture voids, and 100 cleavage facets were manually measured for each of the six material conditions. Several grains of each type (small and large) were characterized in each material to determine the local Y concentration variations with grain size and Y_2_O_3_ content in material composition. EDS was conducted at 20 keV of acceleration voltage to excite ZrKα and YKα lines.

X-ray diffraction (XRD) studies of as-sintered specimens were conducted on a DRON-4.07M diffractometer (Bourevestnik, St Petersburg, Russia). Procedures of indexing, refinement of the profile and structural parameters, and evaluation of the phase weight fractions were carried out by using the WinCSD software package (WinCSD, https://www.wincsd.eu/, accessed on 20 February 2021). The following marking of the ZrO_2_ phase and reference codes were used: t—tetragonal (COD ID 2300612), m—monoclinic (COD ID 1528984), and c—cubic (COD ID 2101234).

## 3. Results

### 3.1. Grain Structure

**Figure 1:**
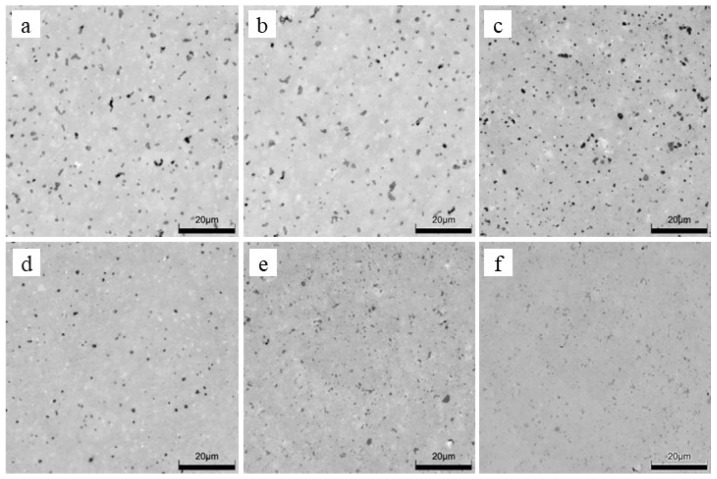
Optical images of non-etched microstructure in the studied materials containing (**a**) 3%, (**b**) 4%, (**c**) 5%, (**d**) 6%, (**e**) 7%, and (**f**) 8% Y_2_O_3_.

**Figure 2:**
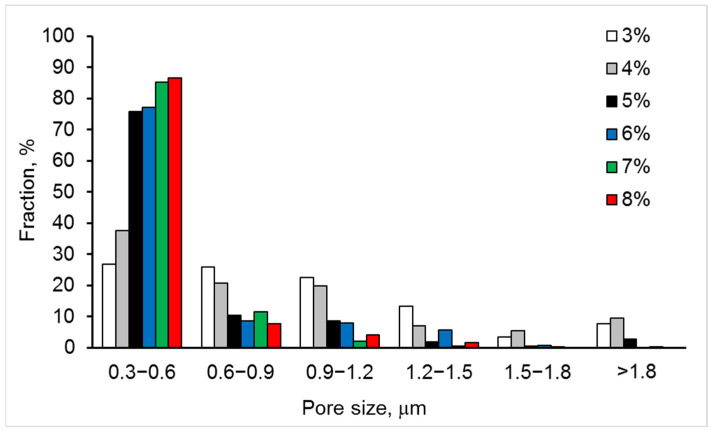
Pore size distributions for six studied materials with 3–8% Y_2_O_3_.

Optical images of the non-etched microstructure in the studied ceramics exhibit a clear dependence of an average pore size on yttria content; namely, the average pore size decreases with an increase in yttria percentage (Figure 1 and Table 1). However, the pore number density and area fraction show dependences with a minimum. The pore size distributions allow for a quantitative characterization of the porosity’s evolution with the yttria content (Figure 2). With an increase in yttria percentage from 3 to 5 mol% (Figure 1a–c), the fraction of 0.6–0.9 μm pores decreased from 25 to 11%, this of 0.9–1.2 μm pores decreased from 22 to 9.5%, and this of 1.2–1.5 μm pores decreased from 13 to 1.5%. At the same time, the fraction of 0.3–0.6 μm pores increased from 26 to 75% (Figure 2). Moreover, negligible fractions for pore-size ranges of 1.5–1.8 μm and >1.8 μm were determined for almost all the studied materials, except for 3YSZ and 4YSZ, where fractions were in the range of 3 to 10%.

In contrast to the abovementioned cases, when the yttria percentage increases from 6 to 7 mol% (Figure 1d,e), the 0.9–1.2 μm pore fraction steeply decreases from 7 to 2%, and the 1.2–1.5 μm pore fraction decreases from 5.5 to 0.5%, while an increase in 0.3–0.6 μm and 0.6–0.9 μm pore fractions from 76 to 84.5% and from 7.5 to 10.5%, respectively, can be seen (Figure 2).

Special attention should be paid to the material with the yttria content increased to 8 mol% (Figure 1f). At these conditions, according to a number of works [37,38,39,40], a completely stabilized cubic zirconia structure can be obtained. This means that, regardless of a sintering mode, this material is disposed to have a higher percentage of stabilized phases, i.e., cubic and/or tetragonal ones. In our case, for 8YSZ ceramics, a pattern of pore size distributions showing fractions of 86%, 8%, 4%, and 1.5% for pore size ranges of 0.3–0.6 μm, 0.6–0.9 μm, 0.9–1.2 μm, and 1.2–1.5 μm, respectively, is observed (Figure 2). Such a pattern indicates slight phase transition from sintering to room temperature, which does not provide conditions for the formation of large pores. Such results are consistent with the literature data [37,39].

**Figure 3:**
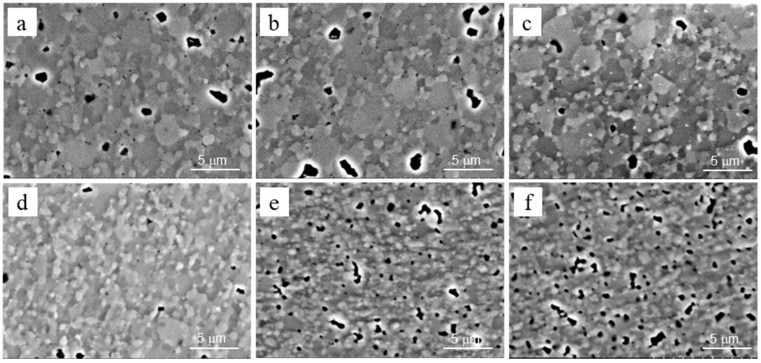
SEM-SE images of microstructures for the studied materials containing (**a**) 3%, (**b**) 4%, (**c**) 5%, (**d**) 6%, (**e**) 7%, and (**f**) 8% Y_2_O_3_.

**Figure 4:**
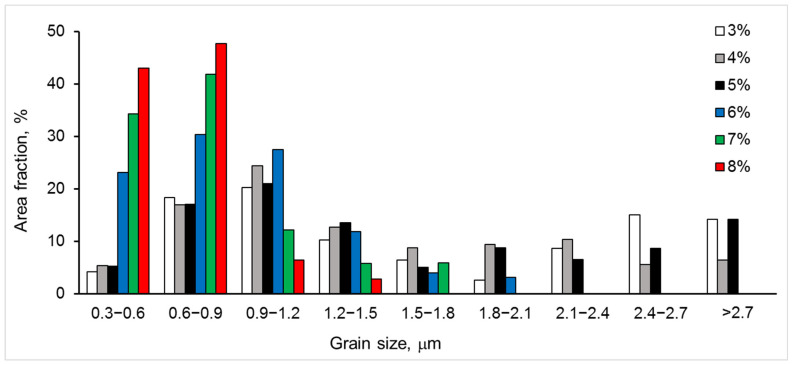
Grain size distributions for six studied materials with 3–8% Y_2_O_3_.

At lower Y_2_O_3_ concentrations of 3–5%, the grain structure showed a distinct bimodality of grain size (Figure 3 and Figure 4). However, the bimodality gradually decreased with an increase in Y_2_O_3_ content.

In terms of the average values of measured microstructural components, with an increase in Y_2_O_3_ content from 3 to 8%, we observed the following:

- The average grain size decreased (Table 1) following an increase in the fraction of smaller grains and a decreased in the fraction of larger grains (Figure 4);
- The grain structure became more homogeneous (notice the bimodality of grain size distributions for 3–5% Y_2_O_3_ and its disappearance for 6–8% Y_2_O_3_);
- The average size of the pores decreased by more than 2 times (Table 1).

### 3.2. Chemical Analysis and Phase Balance

**Figure 5:**
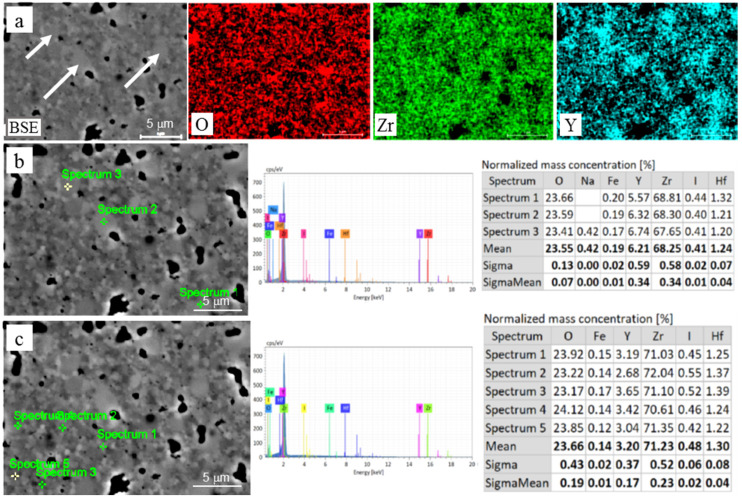
Chemical analysis of 3YSZ material: (**a**) SEM–EDS maps for O, Zr, and Y, showing grains enriched in Y (white arrows); quantitative analysis of (**b**) coarse and (**c**) fine grains with corresponding EDS spectra and concentration tables.

**Figure 6:**
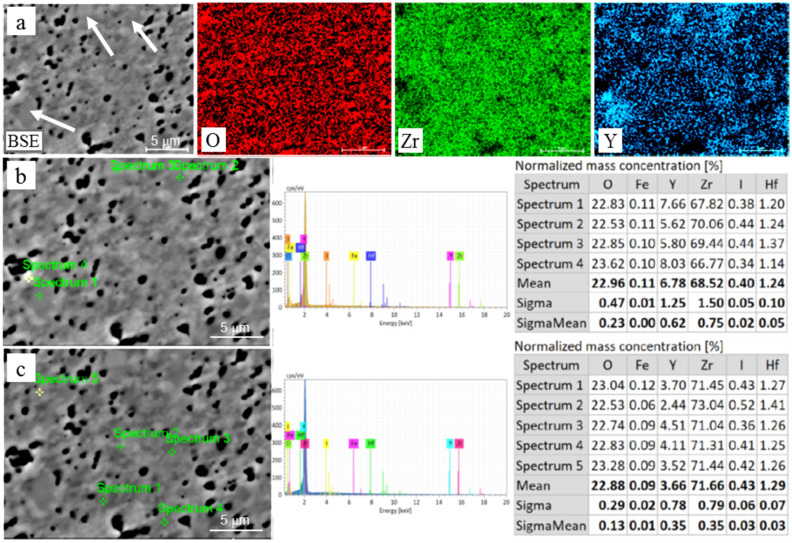
Chemical analysis of 8YSZ material: (**a**) SEM–EDS maps for O, Zr, and Y, showing grains enriched in Y (white arrows); quantitative analysis of (**b**) coarse and (**c**) fine grains with corresponding EDS spectra and concentration tables.

The SEM–EDS mapping showed an inhomogeneous distribution of Y in the studied materials. Thus, the grains that were larger in size were enriched in Y (Figure 5 and Figure 6). Pure ZrO_2_ undergoes a phase transformation during cooling from a cubic crystal structure (above 2370 °C to melt at 2690 °C) to tetragonal (2370–1173 °C) to monoclinic (below 1173 °C) [37,39,41,42]. Increased Y_2_O_3_ concentrations stabilize higher-temperature crystal structure types to room temperature. Therefore, the observed variations in Y_2_O_3_ allow us to expect two or three phases in the studied materials.

**Figure 7:**
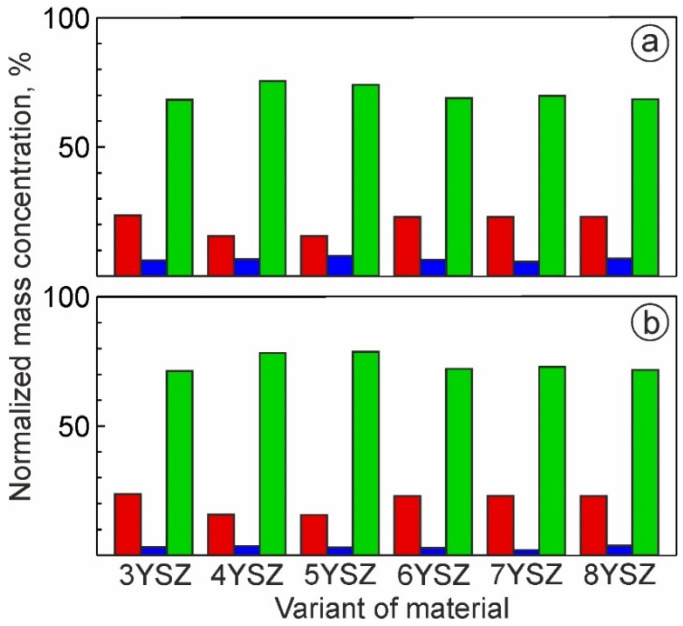
Normalized mass concentration of oxygen (red bars), yttrium (blue bars), and zirconium (green bars) for six studied materials with 3–8% Y_2_O_3_: (**a**) high yttrium content in large grains and (**b**) low yttrium content in small grains.

**Figure 8:**
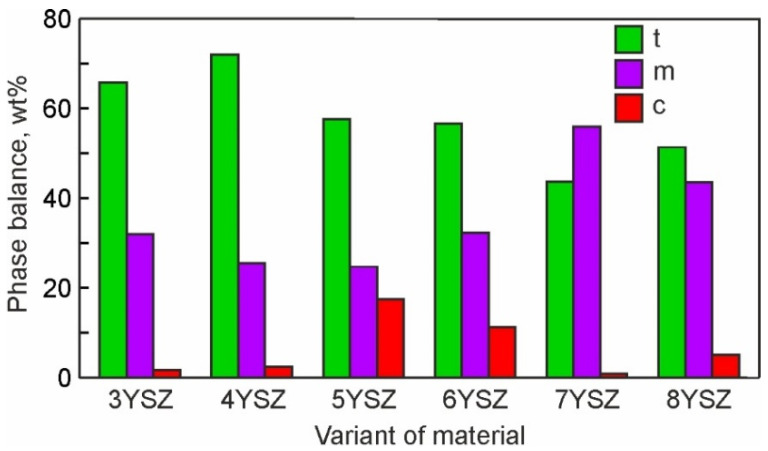
Phase balance for six studied materials with 3–8% Y_2_O_3_. Notation: t is tetragonal, m is monoclinic, and c is cubic ZrO_2_.

Normalized mass concentrations of oxygen (red bars), yttrium (blue bars), and zirconium (green bars) are presented in Figure 7, with respect to the bulk yttria content in the studied ceramics. At least three dependences related to these elements can be observed on the graph: (i) the oxygen concentration decreases and the yttrium percentage increases with an increase in yttria content from 3 to 5%; (ii) the oxygen content is almost equal to this of yttrium with yttria content varying from 6 to 8%; and (iii) both the oxygen and yttrium concentrations change suddenly with the yttria content in the bulk material composition varying from 5 to 6 mol%—these allow us to assume a global transition from one to another mechanism of phase stabilization. To confirm this assumption, the phase balance variation with yttria content in the studied materials is plotted in Figure 8 on the bases of the XRD analysis [33,34]. The tetragonal phase (t-ZrO_2_) showed the maximum concentration at 4% yttria. With a further increase in the yttria content to 5%, a steep decrease in the t-ZrO_2_ phase fraction was observed. In contrast, the cubic phase (c-ZrO_2_) fraction showed its maximum at 5% yttria. With a further increase in yttria content to 6–8%, an increase in the c-ZrO_2_ fraction did not take place, although it might be expected due to a deeper stabilization of ZrO_2_ with the Y_2_O_3_ content. This can be related to the segregation of Y_2_O_3_ during powder mixing, leading to the formation of grains with significantly different Y concentrations (Figure 5 and Figure 6). Reduced local concentrations of yttria resulted in weaker stabilization of high-temperature phases; thus, the fraction of low temperature monoclinic phase (m-ZrO_2_) increased at 7–8% of yttria.

### 3.3. Strength and Fracture Analysis

**Figure 9:**
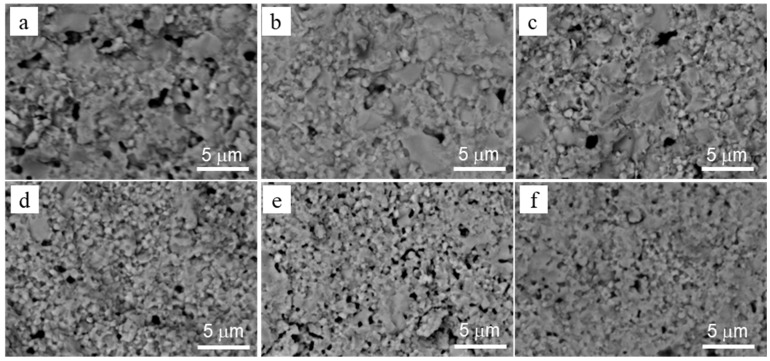
SEM-BSE images of the fracture surfaces for the studied materials containing (**a**) 3%, (**b**) 4%, (**c**) 5%, (**d**) 6%, (**e**) 7%, and (**f**) 8% Y_2_O_3_.

**Figure 10:**
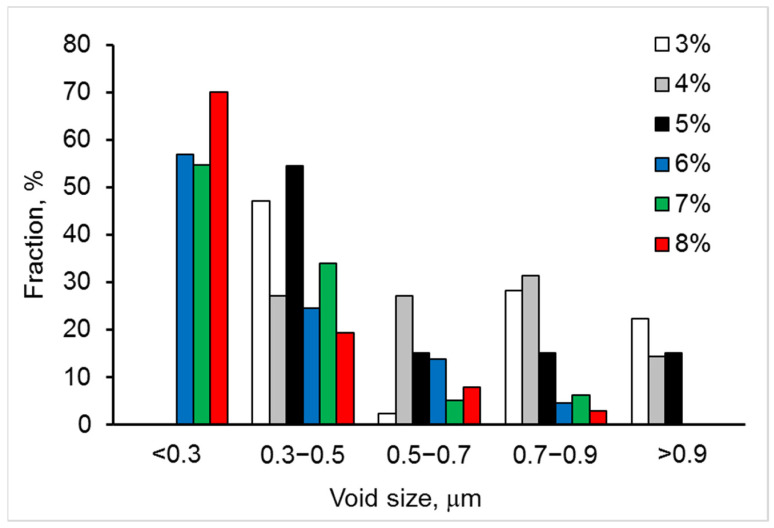
Size distributions of fracture voids for six studied materials with 3–8% Y_2_O_3_.

**Figure 11:**
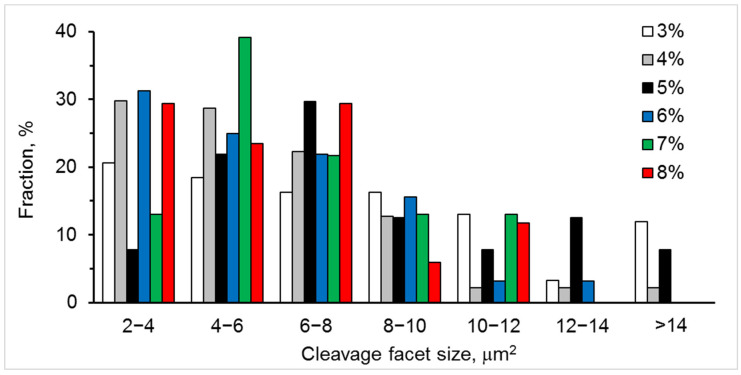
Size distributions of cleavage facets for six studied materials with 3–8% Y_2_O_3_.

SEM images of fracture surfaces for the studied materials are presented in Figure 9. They clearly show the variation in voids’ size and cleavage facet density with yttria content. The 6YSZ, 7YSZ, and 8YSZ ceramics exhibited high fractions of small voids (average size < 0.3 µm), relatively lower fractions of larger voids (average 0.3–0.9 μm), and no voids in the >0.9 μm size range (Figure 10). In contrast, the 3YSZ, 4YSZ, and 5YSZ ceramics showed no voids in the <0.3 µm size range, higher fractions of 0.3–0.7 μm voids, and a reasonable amount of >0.9 μm voids. The analysis of cleavage facet size distributions has shown the following (Figure 11): (i) 5%, 7%, and 8% Y_2_O_3_ correspond to maximal fractions of cleavage facets in the 4–6 and 6–8 µm^2^ size ranges; (ii) 3% and 5% Y_2_O_3_ correspond to maximal fractions of cleavage facets in the 12–14 and >14 µm^2^ size ranges; and (iii) 4% and 6% Y_2_O_3_ correspond to high fractions of cleavage facets in the 2–4, 4–6, and 6–8 µm^2^ size ranges.

In terms of average sizes of fracture voids and cleavage facets, the following dependences were discovered with an increase in Y_2_O_3_ content from 3 to 8%:

- The average size of fracture voids decreased by 2 times (Table 1), the standard deviation of size decreased by 3.5 times, and the number density of voids increased by 3.8 times—all of these mean that the fracture became more homogeneous with an increase in the Y_2_O_3_ content;
- The average size of cleavage facets (associated with a transgranular fracture) decreased by 30%, the number density of cleavage facets decreased by 5.4 times, and the transgranular fracture area decreased by 6.8 times—all of these indicated that the microstructure became finer, and cohesion between grains increased sufficiently to stimulate the fracture propagation along the boundaries of fine-grained agglomerates, except for occasional transgranular fracture through the larger grains with an increased Y content.

**Figure 12:**
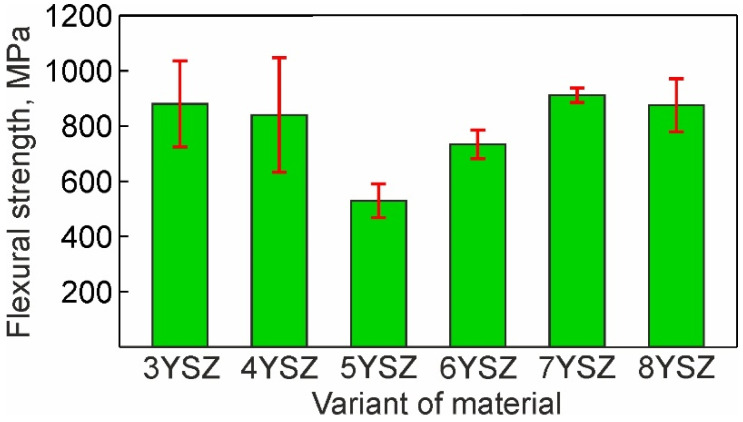
Flexural strength of six studied materials with 3–8% Y_2_O_3_.

The observed variations in fracture voids and cleavage facets are in line with the flexural strength dependence on yttria content (Figure 12). The minimum strength of 5YSZ ceramic corresponds to the relatively high fraction of coarse fracture voids (>0.9 μm) and cleavage facets (>12 μm^2^). The 7YSZ ceramic, having the highest strength, exhibited a peak fraction of the smallest voids (<0.3 μm) and small cleavage facets (4–6 μm^2^).

The YSZ ceramics was shown to contain comparatively large areas of the m-ZrO_2_ phase distributed more or less uniformly in the fine-grained t-ZrO_2_ phase [33]. Therefore, dimensions of the microstructure constituents should be taken into account when analyzing a relation between strength and chemical composition.

Comparing the strength, phase balance, pore and grain size, and void and cleavage facet size distributions for each of the studied materials, the following can be stated:

▪ The flexural strength dependence on yttria content (Figure 12) directly follows the variation in fraction of the monoclinic phase m-ZrO_2_ and inversely follows this of the cubic-phase c-ZrO_2_ (Figure 8). With the yttria percentage increasing from 3 to 5%, the strength decreases following a decrease in the fraction of m-ZrO_2_ and an increase in the fraction of c-ZrO_2_. The peak flexural strength for 7YSZ ceramics corresponds to the maximum of m-ZrO_2_ fraction and minima of c-ZrO_2_ and t-ZrO_2_ fractions;
▪ Large cleavage facets (>12 µm^2^) observed in 3YSZ–5YSZ materials (Figure 11) are probably related to the coarse-grained t-ZrO_2_ and c-ZrO_2_ phases (Figure 4) [40,43], which are enriched in Y (Figure 5b) and show higher hardness and strength than m-ZrO_2_ [41,44,45];
▪ A maximal fraction of cleavage facets in the 6–8 µm^2^ size range observed in 5YSZ corresponds to the maximum of the partially stabilized c-ZrO_2_ phase fraction and the minimum of the m-ZrO_2_ phase fraction; this material has the lowest strength among the studied ceramics;
▪ The maximal fraction of small 4–8 µm^2^ cleavage facets in 7YSZ and this of 2–8 µm^2^ facets in 8YSZ (Figure 11) correspond to the peaks of minimal grain size in the 0.3–0.9 µm size range (Figure 4) and the maximum fraction of m-ZrO_2_ phase (Figure 8); 7YSZ showed the highest strength among the studied ones due to the high cohesion between fine grains of both the t-ZrO_2_ and m-ZrO_2_ phases.

The porosity is suggested to affect the mechanical behavior of YSZ. Thus, with an increase in the Y_2_O_3_ content, the fraction of smaller pores (0.3–0.6 μm) in the original sintered microstructure increased (Figure 2), and this corresponds to the high flexural strength observed in 7YSZ and 8YSZ ceramics.

### 3.4. Fracture Micromechanisms

**Figure 13:**
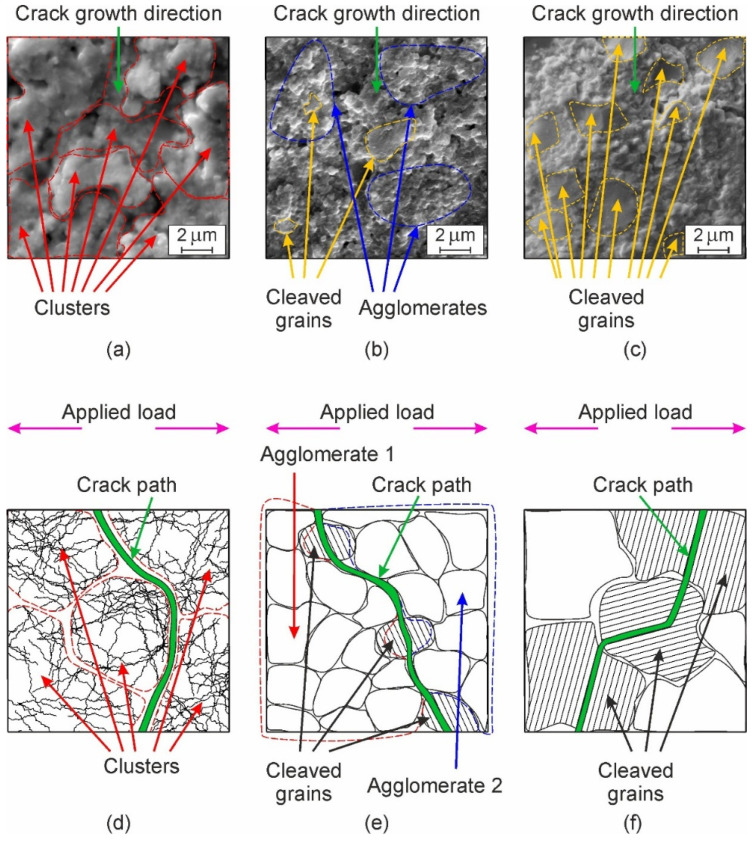
Microstructure evolution in ceramics in relation to their crack-growth resistance: (**a**–**c**) SEM images of characteristic fracture micromechanisms and (**d**–**f**) modeling diagrams for corresponding micromechanisms; the diagrams (**d**–**f**) exhibit crack propagation paths for characteristic fracture patterns under load applied in the direction shown by arrows; the arrows also indicate some microstructure components (cleaved grains, clusters, and agglomerates of fine grains).

The discussed peculiarities of mechanical behavior in the studied materials allow us to generalize the principal mechanisms of their structural strength development. SEM images of fracture surfaces (Figure 13a–c) illustrate the three mechanisms of fracture evolution. If the sintering mode does not promote complete recrystallization and formation of the fine-grained microstructure, clusters of partially sintered particles of the initial powder mixture are formed (Figure 13a). They have weak cohesion to each other, resulting in the comparatively low crack-growth resistance of this microstructure variant [33]. This fracture micromechanism is depicted in Figure 13d and is characterized by the crack propagation path following the pores between the particles and weak particle–particle interfaces. The arrows in Figure 13a indicate clusters which are circumflexed by a crack, while most parts of the clusters themselves remain unbroken. The second fracture micromechanism operates in fine-grained microstructures with high cohesion between completely recrystallized grains (Figure 13b). Recrystallisation promotes the formation of agglomerates of fine grains with a high bond strength, providing an increased bulk strength to the whole material. In this case, a crack propagates along the boundaries of the fine grains or their agglomerates, and cleavage may occasionally take place through the grains located ahead of the crack front (Figure 13b,e). The third fracture micromechanism operates in coarse-grained microstructures (Figure 13c) formed at higher temperatures and longer sintering times, leading to intense grain growth, while the cohesion between the large grains remains at a high level. This provokes the intense cleavage fracture of large grains under loading, especially when cleavage planes having the weakest bonds coincide with the crack propagation plane (Figure 13c,f).

The second fracture mechanism may be considered as the most favorable, as in this case, the crack path undergoes multiple branching occurrences and changes of direction along the boundaries of the fine grains and their agglomerates. This slows down the speed of fracture propagation and improves the strength and toughness. To stimulate operation of the second fracture mechanisms, a fine-grained microstructure with a strong grain boundary cohesion should be obtained. This can be achieved via a careful selection of the ceramic composition (dopant type and content) and the processing parameters (sintering temperature and time).

## 4. Conclusions

The analysis of the microstructure, strength, and fracture behavior carried out in this work for ZrO_2_ ceramics stabilized with 3–8 mol% Y_2_O_3_ (YSZ) and sintered at 1550 °C for 2 h resulted in the following conclusions:

1. With an increase in Y_2_O_3_ content, the average sizes of grains, pores, fracture voids, and cleavage facets all decreased. In addition, the size distributions of all the studied microstructural features became narrower. This indicates the formation of a more homogenous microstructure with an increase in Y_2_O_3_ content.
2. The minimum flexural strength observed for 5YSZ ceramic is associated with the maximum fraction of the cubic ZrO_2_ phase.
3. The highest level of flexural strength was shown for a fine-grained microstructure in the 7YSZ ceramic containing mainly the monoclinic and tetragonal ZrO_2_ phases.
4. Fracture studies suggested the most favorable for practical applications fracture micromechanism operating in fine-grained microstructures with high fraction of the monoclinic ZrO_2_ phase. In this case, the crack propagates along the boundaries of fine grains and their agglomerates, although occasionally cleavage takes place through larger grains, which may be enriched with Y and exhibit a partially stabilized tetragonal or cubic crystal structure.

## Figures and Tables

**Table 1:** Average parameters of microstructure and fracture surface.

% Y_2_O_3_	Average Grain Size, mm	Pores			Fracture Voids		Cleavage Facets		
		Average Size, mm	Number Density, mm^−2^	Area Fraction, %	Average Size, mm	Number Density, mm^−2^	Size, mm^2^	Number Density, mm^−2^	Area Fraction, %
3	1.0 ± 0.5	0.91 ± 0.56	0.0328	2.9	0.67 ± 0.38	0.039	8.0 ± 4.9	0.043	34
4	1.0 ± 0.5	0.90 ± 0.69	0.0287	2.9	0.67 ± 0.29	0.032	5.8 ± 3.0	0.044	25
5	1.0 ± 0.5	0.52 ± 0.42	0.0466	1.6	0.58 ± 0.29	0.031	8.5 ± 4.1	0.030	25
6	0.7 ± 0.3	0.49 ± 0.28	0.0200	0.5	0.36 ± 0.14	0.030	6.1 ± 2.9	0.015	9
7	0.7 ± 0.2	0.41 ± 0.21	0.0459	0.8	0.34 ± 0.13	0.090	6.4 ± 2.6	0.011	7
8	0.6 ± 0.2	0.41 ± 0.19	0.0520	0.8	0.32 ± 0.11	0.147	6.0 ± 2.6	0.008	5

## Data Availability

All the supporting and actual data are presented in the manuscript.
